# The effects of the use of platelet-rich plasma gel on local recurrence in an animal model of human fibrosarcoma

**DOI:** 10.1186/s13027-019-0237-6

**Published:** 2019-08-28

**Authors:** Antonio Barbieri, Sabrina Bimonte, Giovanna Loquercio, Domenica Rea, Marco Cascella, Annamaria Anniciello, Antonio Luciano, Giuseppe Palma, Gaetano Di Costanzo, Azzaro Rosa, Pasquale Giuliano, Claudio Arra

**Affiliations:** 1 Animal Facility, Istituto Nazionale Tumori -IRCCS - Fondazione “G. Pascale”, “Fondazione G. Pascale”, Via Mariano Semmola, 80131 Naples, Italy; 2 Division of Anesthesia and Pain Medicine, Istituto Nazionale Tumori – IRCCS – “Fondazione G. Pascale”, Via Mariano Semmola, 80131 Naples, Italy; 3 S.S.D. Virology and Molecular Biology, Istituto Nazionale Tumori, IRCCS Fondazione G. Pascale, Via Mariano Semmola, 80131 , Naples, Italy; 4 Division of Pathology, Istituto Nazionale Tumori - IRCCS - Fondazione “G. Pascale”, Via Mariano Semmola, 80131 Naples, Italy; 5 SSD Medicina Trasfusionale, Istituto Nazionale Tumori, IRCCS Fondazione G. Pascale, Naples, Italy; 6 Cirugía Plástica, Estética y Reparadora, Hospital Quironsalud Valencia, Valencia, Spain

**Keywords:** Platelet-rich plasma gel, Fibrosarcoma, Local recurrence, Microsurgery

## Abstract

**Background:**

Platelet-rich-plasma (PRP) is largely used, thanks to its properties, as wound therapy after surgical resection. Several studies and clinical findings have demonstrated that the PRP can accelerate the regeneration and the repair of tissues through the action of the platelet-derived growth factors.

**Material and methods:**

Our study aimed to investigate the effects of PRP-gel on the rate of tumor relapse by using a mouse model of Human Fibrosarcoma (HF). The radical resection of tumors of mice was conducted under fluorescence-guidance (FGR) by using MacroFluo microscope, after a primary tumor removal with bright-light surgery (BLS).

**Results:**

It was found that the lesion recurrence and the tumor growth were reduced in mice treated with PRP observed in each group of treatment (50%) after 30 days from tumor excision, respect to controls (without statistical significance; *p* = 0.12). The histopathological and immune-histochemical analysis did not report differences in cellular morphology between the tumors of control and PRP-treated mice.

**Conclusion:**

Our data suggest that PRP-gel, used as an adjuvant treatment for the stimulation of tissue repair and speed up recovery, can impair tumor growth and slow the tumor.

## Background

The clinical management of an imperfect tissue repair caused by surgical or post-surgical cancer treatment, or even congenital or chronic debilitative conditions, is of crucial importance for the structural and the functional reconstruction of tissue and organs [1]. For this reason, it’s safe to point out the needing of improving the scientific knowledge of tissue engineering-based procedures in the reconstructive strategies including the autografts, the stem cells and the use of growth factors [2]. In normal conditions, tissue damage results in the platelet activation and in the secretion of several biologically active proteins and molecules, which are responsible for cellular chemotaxis, proliferation, and differentiation and production of the extracellular matrix that are essential for tissue healing and repair [3].

Approximately 11,000 soft tissue sarcomas (STS) are diagnosed every year in the United States [4–6]. The goal standard of soft tissue sarcomas includes extensive resection with or without adjuvant radiotherapy and/or chemotherapy. At 5 years, the cumulative probability of local recurrence reported in large series ranges from 12 to 28%, 7–11 and the cumulative probability of metastasis ranges from 21 to 40%. Extensive excision can be very invasive and include muscle, tendon, ligament, bone, and joints which often affect limb function. For all these reasons, the presence or the absence of residual cancer cells in the surgical area is a crucial point and determines local recurrence by impairing the prognosis and the survival of patients. Furthermore, local recurrence after the adjuvant therapy is mostly resistant to the treatment, resulting in poor prognosis [7–12]. The use of platelet-rich gel (PRP) obtained by blood centrifugation is an alternative and manageable strategy for the local release of multiple endogenous growth factors for tissue regeneration [13, 14]. After activation PRP releases a pool of growth factors, such as basic fibroblast growth factor [bFGF], insulin-like growth factor-1-1 [IGF-1], transforming growth factor-β [TGF-β], platelet-derived growth factors [PDGFs] and vascular endothelial growth factor [15]. The use of PRP has been reported in many fields of medicine, including the maxillofacial surgery and the treatment of problematic soft tissue ulcers [16, 17]. A growing body of evidence shows that a large number of growth factors (GF) released by the PRP may potentially enhance the tumor progression. Thus, the use of platelet concentrates remains still controversial in the medical literature, even though many applications “in situ” have been developed for regenerative medicine and tissue engineering [18]. In a recent study, Loquercio et al. demonstrated that the use of PRP gel as an adjuvant agent significantly reduces the time required for the bone healing following intra-lesion treatment of benign giant cell tumors [19]. Moreover, PRP gel seems to achieve good functional results without promoting local recurrence.

For these reasons, the present article aims to investigate the possible role of the PRP gel in the enhancement of local recurrence, by performing in vivo studies on a mouse model of Human Fibrosarcoma (HF) cells, HT1080-RFP. Our data suggest that PRP-gel, used as an adjuvant treatment for the stimulation of tissue repair and speed up recovery, can impair tumor growth and slow the tumor local recurrence after radical surgery of fibrosarcoma.

## Methods

### Cell lines

The human fibrosarcoma cell line HT1080 expressing red fluorescent protein (RFP) was purchased from Anticancer Inc. (San Diego, CA), maintained and cultured in DMEM medium with 10% fetal bovine serum (FBS) and 5% penicillin/streptomycin.

### In vivo and ex vivo imaging

Leica Macrofluo Z6 APO (Wetzlar, Germany) with DFC 360 Fx camera is a microscope which uses the fluorescence to evaluate, in oncology mouse models, the tumor and the metastasis generated by injections of fluorescent tumor cells. Fluorescence-guided surgery (FGS) was used, in vivo, to remove a tumor and any lesions in the muscle and tendons and in ex vivo for the evaluation of proximal lymph node metastases or distant metastases.

### Animal studies and treatments

Sixteen Foxnnu/nu female mice (six/eight-week-old) were purchased by Harlan (San Pietro al Natisone, Italy) were housed 8 per cage and maintained on a 12-h light: 12-h dark cycle (lights on at 7.00 a.m.) in a temperature-controlled room (22 ± 2 °C) and with food and water ad libitum. The experimental procedures were followed according to EU Directive 2010/63/EU for animal experiments and with the institutional guidelines of the Italian Ministry of Health Animal Care and Use Committee. After 1-week of acclimation to the housing conditions, mice were injected subcutaneously into the right hind limb with a suspension of HT1080-RFP cells (1 × 106 cells/mouse in 200 μl PBS). After approximatively 10 days, the graft of tumors was assessed by MacroFluo microscope, and the mice were randomized for tumor volume and weight according to Fisher’s exact test in two groups: i) PRP- Group (PRP was applied after tumor excision); ii) CTR Groups (tumors were excised without application of PRP gel). The tumors mass was measured with a digital caliper (2BIOL, Besozzo, Italy). When the average of volume tumors gets to 6 × 6 mm2, lesions were removed. All mice were anesthetized via intraperitoneal injection with Zoletil 100 (Virbac), a combination at 50% of tiletamine and zolazepam. It was used at a dose of 50 mg/kg, by adding atropine sulfate at 0.04 mg/kg and xylazine. The skin was cleaned in aseptic conditions with betadine and then cut with a scalpel. Tumor masses were removed firstly by bright-light surgery (BLS) and then by fluorescence-guided surgery, to accurately remove any residual tumor cells. PRP was applied directly on the surgical wound in mice of PRP-treated group GROUP PRP after tumor removal, before closing it with surgical clips. In mice of the control groups, PRP was not applied and only tumor removal was performed. All mice were observed for 30 days to evaluate tumor relapse and followed for tumor growth. Body weight and general conditions were evaluated. When mice reached the fixed cut-off of tumor volume (1500 mm3), they were euthanized by cervical dislocation. The primary tumors and local recurrence were resected, weighed, and divided into two parts, one frozen and one fixed in formalin and paraffin-embedded for immunohistochemical analysis.

### Platelet-rich plasma (PRP) preparation

PRP-gel was prepared on the day of the surgical excision of tumor in mice by researchers of the Department of Haematology-Oncology and Stem Cell, Transfusion Medicine Unit at Istituto Tumori Fondazione G. Pascale IRCCS, Naples, Italy as detailed below. The allogeneic platelet gel was obtained from the blood of a human donor with a platelet count greater than 200 × 10^3^/μL and negative screening test results. The whole blood was centrifuged at 3500 rpm for 11 min at 22 °C to separate it into red blood cells and PRP. The blood component (thrombin) was produced from the allogeneic whole blood using a “home-made” system. To activate the PRP autologous and to accelerate the gelling process, thrombin allogeneic was prepared by adding calcium gluconate to the platelet-poor plasma (PPP)/PRP, (ratio 0.2:1 ml). After 15–40 min of incubation at 37°, the product was centrifuged at 1800 g for 10–15 min. One milliliter of thrombin-containing supernatant and 0.50 ml ionized Ca++ were added to the previously separated PRP, in a Petri dish (Falcon, Becton Dickinson Labware), and mixed until a gelatinous mixture was obtained. The procedures were performed under a laminar-flow hood (Faster Bio48). During the entire process, the phases and several quality controls were carried out. The platelet concentration in the final mix was 1000 to 1500 × 103/μL.

### Histology and immunohistochemistry

For histological assessment, formalin-fixed and paraffin-embedded 4 μm sections were stained with Hematoxylin and Eosin (H&E) for morphological tissue analysis. Sections of 4 μm were mounted on positively charged glass slides for immunohistochemistry (IHC). Antigen retrieval pre-treatment was performed using heat-induced antigen retrieval (HIER) in EDTA buffer solution (pH 8.0) for 3 times (5 min each) in a pressure cooker at 98 °C. Following, endogenous peroxidase (EP) activity was quenched with 100 μL of Peroxidase Block (RE7101 Novocastra/Leica Biosystems, Milan, Italy) for 10 min at room temperature and, afterward, 100 μL Protein Block (RE7102 Novocastra/Leica Biosystems, Milan, Italy) were applied on each section. Slides were sequentially incubated overnight at 4 °C with Rabbit polyclonal to Ki67 (ab15580, Abcam, Cambridge, UK) diluted 1:200 in PBS (0.01 M PBS, pH 7, 2). After the incubation with the antibody, immunodetection was performed with avidin-biotin-peroxidase complex kit reagents (MACH2; Biocare Medical, Concord, CA) with diaminobenzidine chromogen as the substrate in all cases. Finally, sections were counterstained with hematoxylin and mounted. Between all incubation steps, slides were washed two times (5 min each) in TBS. To test the specificity of staining, a negative control was simultaneously performed for each tissue section by omitting the primary antibody. For the morphological evaluation, the degree of cellular atypia, necrosis, hemorrhages as well as the inflammatory infiltrate and the vascular and perineural invasion were assessed and scored by two independent observers (AMA and DDB) in each specimen and under blinded conditions as it follows: absent (−), mild (+), moderate (++) and severe (+++). The score of Ki67 immunoreactivity was measured as the percentage of positively stained nuclei, referred to the entire section.

### Statistical analysis

Data are presented as the mean ± SD. Differences between-group were assessed by Student’s t-test or one-way analysis of variance (ANOVA) followed by a Bonferroni, as appropriate. Statistical significance was defined as *p* ≤ 0.05.

## Results

### Generation of xenograft fibrosarcoma mouse model and tumor resection

**Fig. 1:**
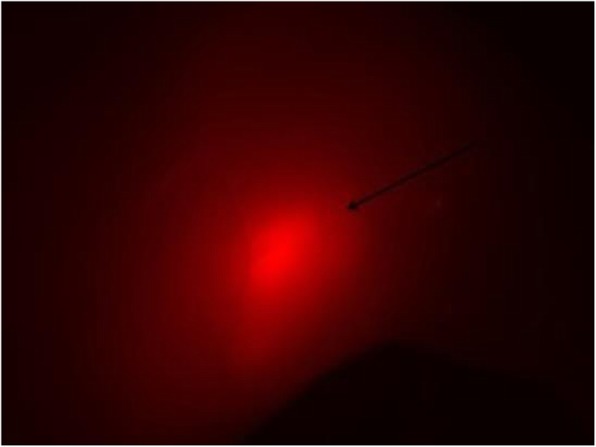
Evaluation of fibrosarcoma engraftment at the fluorescent microscope. The picture shows tumor engraftment ten days after the injection of 1 × 10^6^ HT1080-RFP at the fluorescent microscope. Arrow indicates the tumor visualized by RPF

A xenograft fibrosarcoma mouse model was generated by using HT1080 labeled with red fluorescent protein (RFP) cells (1 X 106) subcutaneously injected into the right hind limb of nude mice. A Xenograft tumor grown between subcutaneous and quadriceps femur and the engraftments were evaluated under fluorescent microscopy as showed in Fig. 1.

**Fig. 2:**
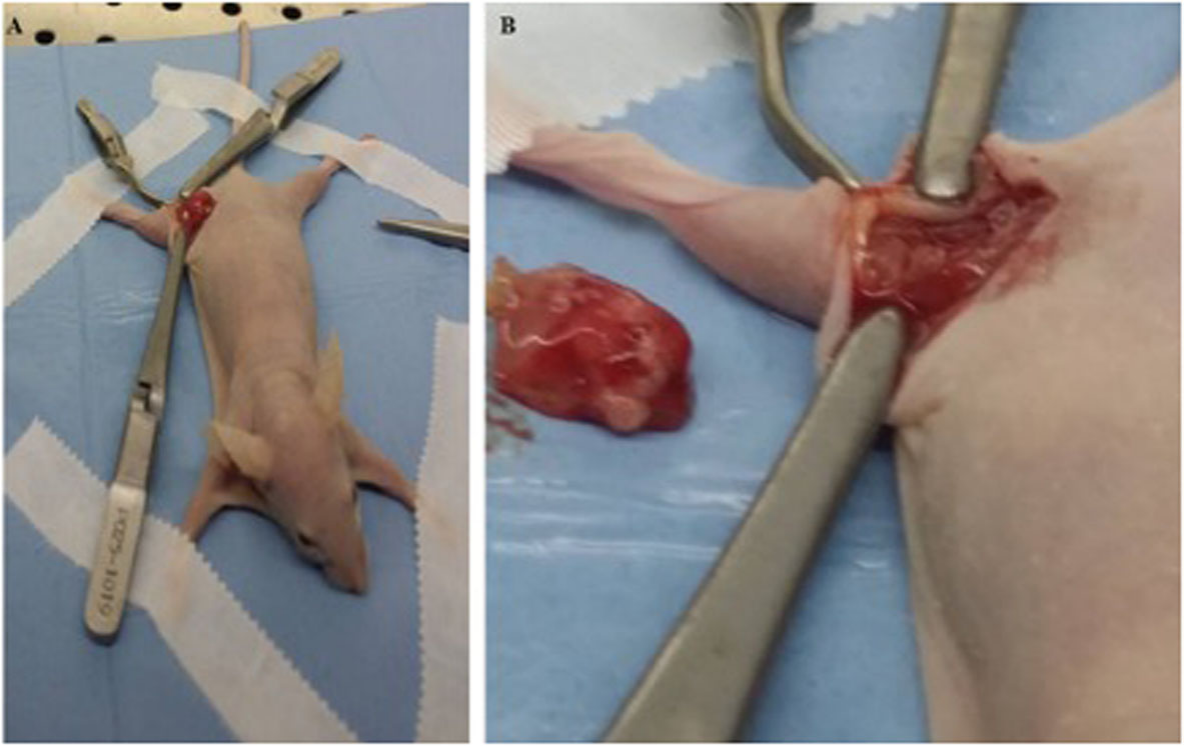
Bright Light Surgery in the xenograft mouse model of fibrosarcoma**. a**-**b** Pictures show the Bright-light surgery (BLS) procedure to remove tumours from a mouse model of fibrosarcoma

After 10 days from the tumoral cells injections, the nodules reached a volume of 100 mm3, assessed by measurements obtained by using the digital caliper and by fluorescent microscopy with MacroFlou. At this time, the tumors were resected. Principal lesions were removed by bright-light surgery (BLS), (Fig. 2a-b).

**Fig. 3:**
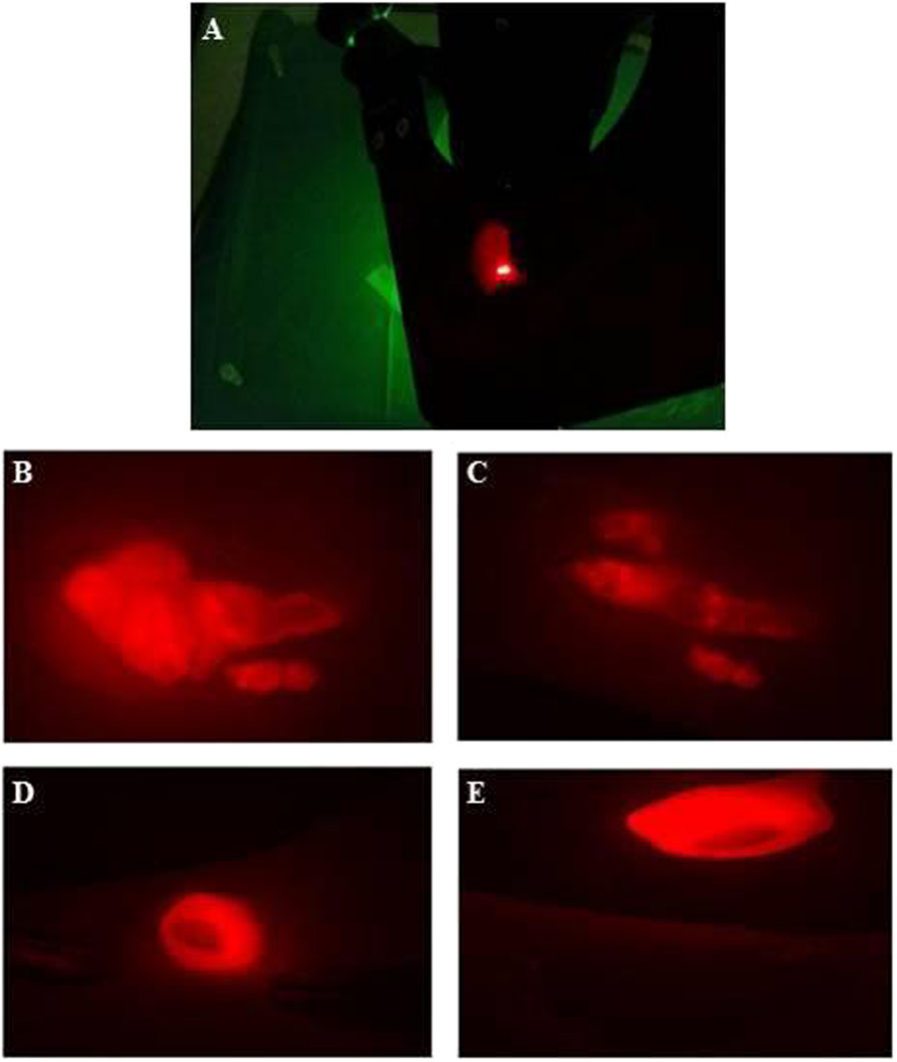
Evaluation of tumor mass at fluorescent microscope and Fluorescence guided surgery in the xenograft mouse model of fibrosarcoma. **a** Representative picture of the evaluation of tumor mass under MacroFluo. **b-e,** Representative pictures of fluorescence guided surgery (FGS) before and after tumors removal with bright light surgery (BLS). In one mouse (**e**) is clearly evident the presence of residual cells infiltrating into the quadriceps muscle after the tumor removal by BLS while in another mouse (**d**) FGS indicates a precise resection of tumor mass without any residual cells in the surgical wound

Since the tumors were extremely aggressively due to their invasions into the femoral quadriceps, the muscles and in some cases into the femoral bone tissue, fluorescence-guided surgery (FGS) with the Macroflou which allowed to visualize the fluorescence of the HT1080-RFP cells (highlighted by red fluorescence), was used to remove these tumour’s infiltrations as showed in Fig. 3a-e.

**Fig. 4:**
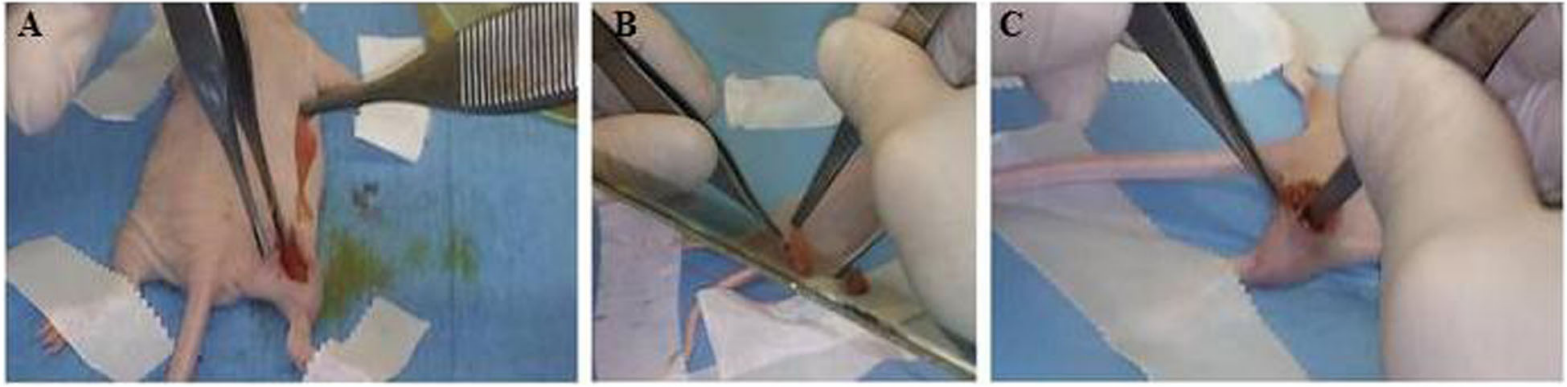
PRP gel’s application on the surgical wound after tumor’s removal in the xenograft mouse model of fibrosarcoma**. a-c.** Representative images of PRP gel’s application after tumor mass removal on the surgical wound

By using this technique an accurate tumor resection can be obtained by carefully eliminating the residual cells in the surrounding tissues to reduce the risk of tumor local recurrence. PRP-gel was applied in a total of 8 mice (PRP-treated mice) for 1 month after removal of tumours (Fig. 4a-c).

### Evaluation of the tumor relapse in xenograft mice after treatment with PRP gel

**Fig. 5:**
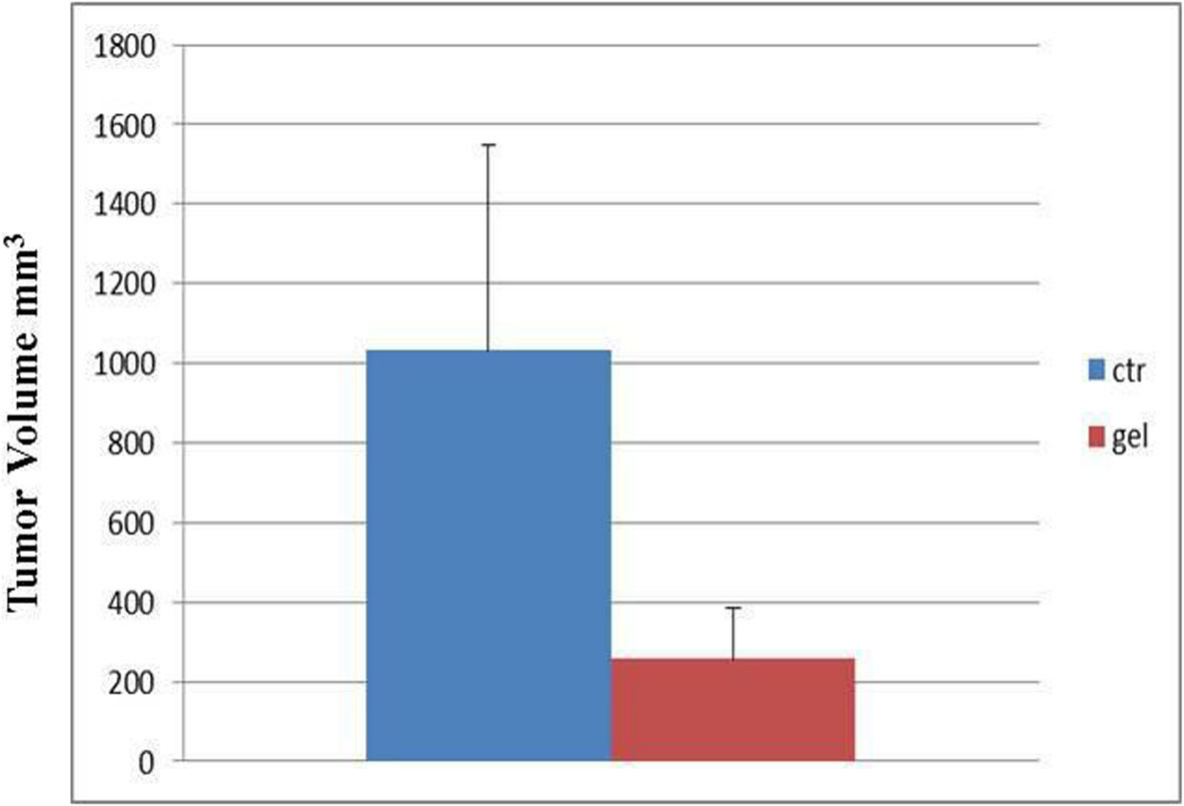
Evaluation of the tumour growth of relapse in xenograft mice after tumour removal and treatment with PRP-gel**.** Tumour volume of mice treated with PRP was reduced (303 mm^3^), although without statistical significance (*p* = 0.12) respect to that observed in not-treated mice (1033 mm^3^)

One month after tumor resection by FGS and after the application of PRP-gel on a total of 8 mice, tumor relapse and tumor growth measurements were assessed in both groups of mice. Tumor volume of mice treated with PRP was reduced (303 mm^3^), although without statistical significance (*p* = 0.12) respect to that observed in not-treated mice (1033 mm^3^) (Fig. 5).

Tumour local recurrence (37, 5%) was reduced in mice-treated with PRP (*n* = 3 over *n* = 8), respected to that observed in controls (50%; *n* = 4 over n = 8 mice). It’s important to underline that, differently from mice treated with PRP, tumor local recurrence was already detected after 13 days from FGS in controls (n = 4), suggesting that PRP-gel can retard tumor local recurrence (data not shown). Furthermore, PRP-gel was able to retard tumor growth since, after 1 month from its application and FGs, none of the mice treated with PRP reached the cut-off (1500 mm3), differently from controls in which one mouse reached the cut-off and was euthanized. Finally, no local or distant metastases were detected in both groups of mice.

### PRP gel is not able to induce changes in fibrosarcoma cellular morphology

**Fig. 6:**
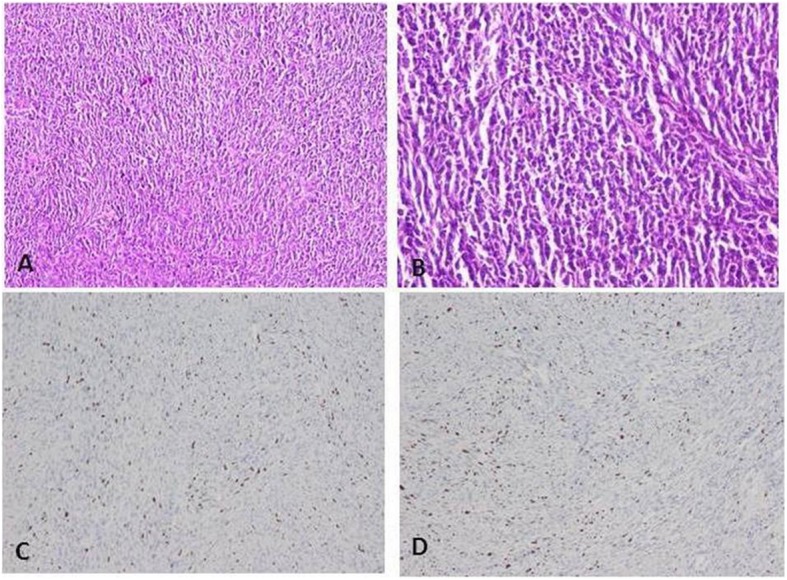
Histological and immuno-histochemical analysis with Ki67 on tumor sections of the xenograft mouse model of fibrosarcoma**. a-b**. Long and short interlacing streams and bundles of spindle cells are separated by the fibromatous matrix. Cells have indistinct borders, a slightly eosinophilic fibrillar cytoplasm and oval to fusiform nuclei with finely stippled chromatin. Hematoxylin and eosin, original magnification 10x for **A** (PRP-treated group) and 20x for B (controls). **c-d** Ki67 immunostaining in the PRP-gel-treated group (**c**) and group control (**d**). Hematoxylin counterstain, original magnification 10x

To evaluate the cellular morphology of fibrosarcoma tumor sections, histological and immunohistochemical analysis with Ki67 antibody was performed on tumors from controls and PRP-treated mice. Microscopic evaluation revealed an expanding, not encapsulated, infiltrative and moderately cellular neoplasm composed of long and short interlacing bundles of spindle cells separated by a slightly eosinophilic and fibrillar fibro-myxomatous matrix. Cells had indistinct borders, a small amount of eosinophilic cytoplasm, oval to fusiform nuclei with finely stippled chromatin and 1–3 prominent magenta nucleoli. Cellular atypia such as anisokariosis and anisocytosis were generally mild to moderate in both groups (Fig. 6a-b). Immunoreactivity for ki67 was detected in the nuclei of neoplastic cells in tumor tissue from control and PRP-treated mice. Peripheral and perivascular aggregates of small lymphocytes, plasma cells and neutrophils were rarely observed (two out of ten cases). (Fig. 6c-d).

## Discussion

Platelets play a fundamental role in the wound healing and in the tissue regeneration by secreting intracellular substances and growth factors that regulate cell proliferation, cell differentiation, angiogenesis, matrix deposition, and tissue remodeling [18, 20].PRP is an autologous source of platelet-derived growth factor and transforming growth factor-beta that is obtained by sequestering and concentrating platelets by gradient density centrifugation. The application of PRP for tissue regeneration and repair is broadly accepted in different medical areas such as dermatology, orthopedics, maxillofacial surgery and in pain medicine [18, 21]. PRP application in oncologic surgery is still controversial and it’s currently arousing great interest from the scientific community [22, 23]. The beneficial effect of PRP after a tumor excision is yet to be fully elucidated; platelet secretory proteins include growth factors that stimulate angiogenesis [20, 23], hence they could promote tumor proliferation and eventually a local recurrence. As previously demonstrated in a small clinical trial, [19]. the use of platelet gel as an adjuvant significantly reduces the time required for bone healing following intra-lesion treatment of benign giant cell tumors and achieves good functional results without promoting local recurrence. In light of these considerations, the present work aimed to further validate the application of PRP gel as a valiant adjuvant in the wound healing and repair and, at the same time, investigate its possible role in the enhancement of local recurrence. Our results showed that mice from both experimental groups developed local recurrence after total excision of the tumor, even though differences in tumor growth rate were observed. Specifically, time to local recurrence was longer in mice of PRP treated group compared to controls. Moreover, the weight and dimension of tumor recurrence were interestingly, but not significantly, lower in PRP treated group compared to controls. Furthermore, according to our results, PRP gel apparently did not negatively promote morphological and behavioral changes in tumor cells as confirmed by Ki67 analysis and other histologic parameters. Unfortunately, this study has some important limitations. First, the local recurrence occurred also in mice treated with the application of PRP-gel on the surgical wound. Even though we didn’t observe an increase in tumor malignancy and aggressiveness, it would be inconsiderate to state that the application of PRP gel is safe and without side-effects in oncologic surgery. Second, the number of animals was quite small, so it is elusive to completely evaluate the outcome of the treatment. For this reason, further studies with a larger number of cases coupled to different clinical and experimental “scenarios”, are necessary to better understand the benefits of PRP gel application in oncological surgery. Regardless, in our opinion, this study offers certain innovation whereas a focus on the application of PRP as an adjuvant in oncologic surgery is still lacking in the medical literature.

### Conclusion and future perspectives

In a view of future research, we would like to share our experience and promote the value of this mouse model because it represents a suitable model for translational studies since it mimics tumor relapse behavior and allows studying more deeply the underlying mechanism. Xenograft fibrosarcoma model in addition to FBS permits a more accurate resection of the tumor and complete removal of residual cancer cells in the surgical area compared to BLS. Tumor recurrence and metastases are a major cause for poor survival rates, even though the patients (mainly with advanced cancer) undergo a complete tumor resection and chemotherapy treatment [24]. Thus, this mouse model could be considered as a valuable fashion tool for the analysis of local recurrence and, moreover, for testing new drugs. The persistence of highly chemotherapy-resistant cancer stem cells (CSCs) is a primary cause of relapse and metastasis. Although highly efficacious chemotherapies suppressing subpopulations of CSCs in various tissue-specific cancers are available, relapse and locale recurrence are still common in patients. We think that in the next future is necessary to identify the mechanisms underlying metastasis, the development of local recurrence and drug-resistance associated with CSCs to find a more suitable therapy for tumor relapse [24]. For these reasons, this mouse model allows gaining a deeper knowledge of the mechanisms involved in tumor relapse.

## Data Availability

Data generated or analyzed during this study are included in this published article.
